# Knockout of the OsNAC113 Transcription Factor Causes High Salt Resistance in Rice

**DOI:** 10.3390/plants14233673

**Published:** 2025-12-02

**Authors:** Bo Wang, Xin Zhao, Qian Wang, Chao Xu, Xin Qi, Yinxia Zhu, Mingjie Lyu, Yong Wang, Chengbin Chen, Yong Zhang

**Affiliations:** 1 State Key Laboratory of Vegetable Biobreeding, Institute of Germplasm Resources and Biotechnology, Tianjin Academy of Agricultural Sciences, Tianjin 300071, China; wangbo0426mm@163.com (B.W.); zhaoxin2008999@126.com (X.Z.); wangqian881001@126.com (Q.W.); xuchao5131@163.com (C.X.); qixin_taas@163.com (X.Q.); 18322712771@163.com (Y.Z.); lvmingjie_good@163.com (M.L.); 2 College of Life Sciences, Nankai University, Tianjin 300071, China; 3 Integrative Science Center of Germplasm Creation in Western China (Chongqing) Science City, Chongqing Key Laboratory of Tree Germplasm Innovation and Utilization, School of Life Sciences, Southwest University, Chongqing 400715, China

**Keywords:** OsNAC113, salt stress, NAC transcription factor, rice, ABC transporters, *Oryza sativa* L. spp. *japonica*

## Abstract

The plant NAC (NAM, ATAF1/2, and CUC2) transcription factor family plays an important regulatory role in stress response. In this study, we analyzed the rice transcription factor *OsNAC113* and elucidated its tissue-specific characteristics and stress response regulatory mechanisms. qRT-PCR results showed that under laboratory-simulated drought, high salt, temperature stress, and hormone treatments, such as abscisic acid (ABA) and gibberellic acid (GA_3_), the expression level of *OsNAC113* significantly changed, indicating that *OsNAC113* responds to various stress conditions. Targeted creation of the rice (*Oryza sativa* L. spp. japonica) *OsNAC113* (LOC_os08g10080.1) mutant based on the CRISPR-Cas9 genome editing strategy revealed its response to salt stress (200 mM). The growth status and survival rate of the mutant under high-salt stress were significantly higher than those of the wild type. Testing showed that the mutant exhibited increased relative water, chlorophyll, and soluble sugar contents under salt stress than the wild type. The malondialdehyde content in the mutant was lower, and the activities of superoxide dismutase, peroxidase, and catalase were higher than those in the wild type, indicating that the mutant with functional loss caused by knocking out *OsNAC113* had a significantly enhanced tolerance to salt treatment. Using RNA-seq to detect genome-wide changes in OsNAC113 mutant materials under stress, KEGG annotation showed that knocking out *OsNAC113* resulted in regulatory changes in “plant hormone signaling pathway” and “MAPK signaling pathway,” and GO and KEGG annotations showed significant changes in “amino acid transport and metabolism,” “carbohydrate transport and metabolism,” “lipid transport and metabolism,” and “replication, recombination, and repair.” *OsNAC113* may be involved in the response to salt stress by regulating these signaling pathways. Using comparative metabolomic analysis, we further elucidated the function of *OsNAC113* in physiological metabolic pathways. The knockout of *OsNAC113* resulted in changes in various important metabolic pathways in plants, including flavonoid biosynthesis and ABC transporters. Therefore, it is suggested that *OsNAC113* is involved in these metabolic processes and affects their regulation in high-salt environments. These results provide a theoretical foundation and reliable material for the molecular breeding of rice.

## 1. Introduction

Rice is a staple food in China, which exhibits a long history of cultivation and widespread popularity among its population. Currently, over 6% of the global land area is increasingly affected by salt accumulation, which affects plant growth, crop production, and ecosystem balance [1]. Soil salinization is a major abiotic stressor affecting agriculture worldwide. Sodium ions affect the structure and characteristics of soil, indirectly causing damage to plant roots. Under high-salt stress, plant cell walls undergo changes in composition and structure, and enhance polysaccharide synthesis and the expression of related modification genes to maintain cell integrity and improve adaptability to salt stress [2].

Numerous studies have demonstrated the crucial role of NAC transcription factors in plant responses to abiotic stress. The Arabidopsis transcription factor *NAC016* inhibits the transcription of AREB1 through the tricarboxylic acid regulatory loop involving NAP, thereby enhancing the plant’s response to drought stress [3]. The rice NAC transcription factor *ONAC066* is drought-responsive, and various stress treatments can significantly induce its expression of *ONAC066*. Overexpression of *ONAC066* enhances drought tolerance in transgenic rice [4]. *VvNAC17* is a novel grape stress-responsive transcription factor that enhances the sensitivity of transgenic Arabidopsis to abscisic acid (ABA) and improves its salinity, freezing, and drought resistance [5]. The NAC domain transcription factor *GmNAC06* enhances soybean salt tolerance. Combining overexpression technology with the CRISPR-Cas9 system revealed that *GmNAC06* facilitated the accumulation of proline (Pro) and betaine, thereby alleviating or preventing the negative effects of reactive oxygen species (ROS) [6]. The wheat NAC transcription factor *TANAC29* improved the salt and drought tolerance of transgenic *Arabidopsis thaliana*, and overexpressed plants *TANAC29* improved their tolerance to high salt and dehydration, and showed high sensitivity to ABA [7]. Yeast one-hybrid experiments demonstrated that the protein encoded by *PsnNAC090* can bind to ABA-responsive elements (ABRE), and its ectopic expression enhances the salt tolerance and permeability of transgenic tobacco [8]. IbNIEL-mediated degradation of *IbNAC087* regulates jasmonic acid-dependent salt and sweet potato drought resistance [9].

A total of 158 NAC transcription factors have been identified in rice; however, only a limited number have been investigated or demonstrated to have functions [10]. Therefore, investigating additional NAC transcription factors is necessary. Effective strategies for molecular breeding crops involve analyzing the tissue-specific and stress-induced expression characteristics of *OsNAC113* combined with CRISPR/Cas9 technology, site-directed mutagenesis of target genes, construction of a rice NAC transcription factor mutant library, and screening NAC transcription factors with notable research potential for further investigation. This study aimed to analyze the rice transcription factor *OsNAC113*, elucidate its tissue-specific characteristics and stress response regulatory mechanisms, and provide comprehensive foundational data and a theoretical framework for further exploration of *OsNAC113*. The acquisition of rice stress-responsive *OsNAC113* functional deletion mutants provides important experimental material for subsequent research. Through a joint omics analysis of the transcriptome and metabolome, *OsNAC113* was systematically analyzed at the gene regulation and cellular metabolic levels.

## 2. Results

### 2.1. Analysis of OsNAC113 Expression Pattern

**Figure 1:**
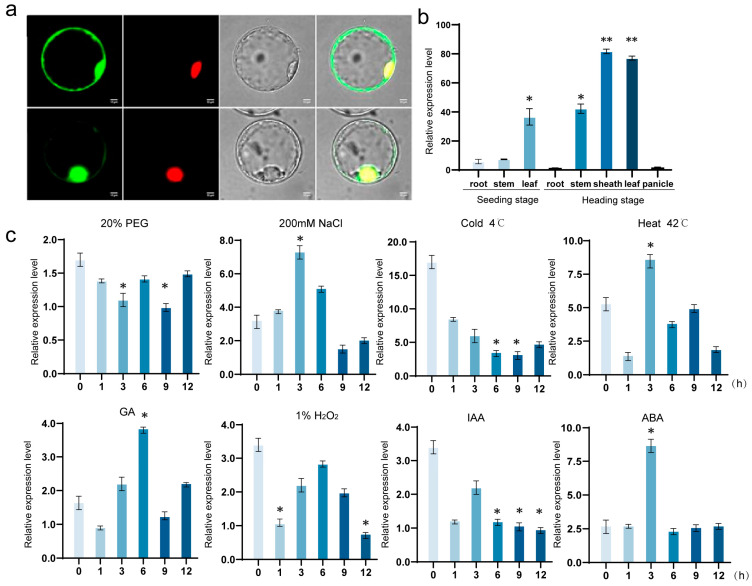
Expression pattern analysis of OsNAC113. (**a**) Subcellular localization of OsNAC113 expression. Green represents organelles, while red represents the nucleus (**b**) Tissue-specific expression pattern of OsNAC113. The *x*-axis represents different sampling positions. Root, sheath, and leaf samples at the seedling stage. Root, stem, sheath, leaf, and panicle samples at the reproductive growth stage. (**c**) Expression levels of OsNAC113 under various abiotic stresses and hormone treatments. Four-week-old seedlings were exposed to the following treatments: cold (4 °C), heat (42 °C), PEG 6000 (20%, *w*/*v*), NaCl (200 mm), H_2_O_2_ (1%), IAA (100 μm), ABA (100 μm), and GA3 (100 μm). The *x*-axis represents unused processing time. Materials were extracted at 0, 3, 6, 9, and 12 h. The relative expression level of OsNAC113 was measured at RT-qPCR at the indicated times. Error bars indicate SE based on three independent biological replicates. * and ** represent significant differences at *p* < 0.05 and *p* < 0.01 compared to WT.

NetPhos-3.1 was used to analyze the phosphorylation sites of the *OsNAC113* encoded protein, which contained only threonine phosphorylation sites (Supplementary Figure S1). We used the expression vector pBWA (V) HS-*OsNAC113*-Glosgfp10509 to determine the specific location of *OsNAC113* expression, where the strong constitutive 35S promoter drives the transcription of the *OsNAC113* gene. Due to the fusion of the *OsNAC113* protein with sGFP, the real-time localization of the *OsNAC113* sGFP fusion protein in living plant cells can be observed through confocal microscopy. Fluorescence microscopy revealed that *OsNAC113* was expressed in the nucleus (Figure 1a). The roots, stems, and leaves of four-week-old wild-type plants, as well as the roots, stems, sheaths, leaves, and panicles of 15-week-old wild-type plants, were collected, and the expression levels of *OsNAC113* in different tissues and organs were detected using RT-qPCR. These results indicated that *OsNAC113* is mainly expressed in leaf-related tissues and organs, possibly through the regulation of leaf photosynthesis and other stress responses (Figure 1b). Expression levels are minimal in the roots and other tissues, and are highly expressed in the sheath at maturity. *OsNAC113* responded to various stresses, among which high-salt stress and temperature changes were more significant in inducing *OsNAC113* expression (Figure 1c). The predicted binding sites for the *OsNAC113* promoter included 14 available sites for Wie: WRKY71OS. The recognition site of ACGTATERD1 appeared eight times, and the recognition sequences of other dehydration-related transcription factors MYBCORE were CNGTTR, MYB2CONSENSUSAT, rd22, and Dof protein (Supplementary Table S3). The Protscale software Expasy predicted that the hydrophilicity of the *OsNAC113* encoded protein was unevenly distributed throughout the peptide chain, with most amino acids located below 0, indicating that the *OsNAC113* protein has strong hydrophilicity, which is consistent with the predicted physicochemical properties (Supplementary Figure S2). According to the TMHMM prediction, the *OsNAC113* encoded protein does not contain transmembrane regions and is an extracellular protein (Supplementary Figure S3).

### 2.2. OsNAC113 Directed Editing Mutant Creation

**Figure 2:**
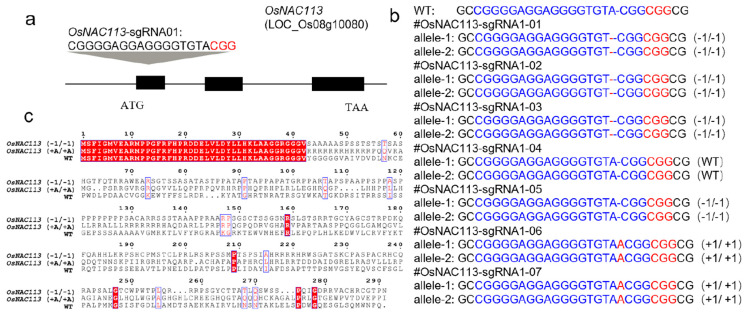
Creating mutants using the CRISPR-Cas9 system. (**a**) Design of the sgRNA site for OsNAC113 exons. (**b**) Sanger sequencing results of osnac113 T0 generation. (**c**) Changes in OsNAC113 mutant protein expression.

*OsNAC113*-sgRNA01 was designed in the first exon sequence, and a targeted knockout expression vector was constructed using a Golden Gate reaction (Figure 2a). PCR amplification of the target sequence and sequencing analysis have been performed to obtain the genotype. After testing, five of seven transgenic positive plants were mutated, with a mutation rate of 71.4%. Specifically, *OsNAC113*-sgRNA01-04 and 05 were wild-type genotypes without any mutations. *OsNAC113*-sgRNA01-01, 02, and 03 were mutant variants lacking one base. *OsNAC113*-sgRNA01-06, 07 were mutants with a single nucleotide A insertion and homozygous mutation, providing material for subsequent experimental screening (Figure 2b). In the mutant *osnac113* (−1 bp/−1 bp), the translation of amino acids was terminated prematurely, and the amino acid sequence translated by the mutant *osnac113* (+A/+A) was significantly different from that of the wild type (Figure 2c). The sgRNAs were designed to predict off-target sites based on the CRISPR-P website. Primers were designed upstream and downstream of the off-target sites for sequence detection to verify the presence of off-target phenomena. Sequencing analysis was conducted on the top 10 most likely off-target sites, and no other cuts were detected (Supplementary Table S4).

### 2.3. Phenotyping Analysis of OsNAC113 Under Salt Stress

**Figure 3:**
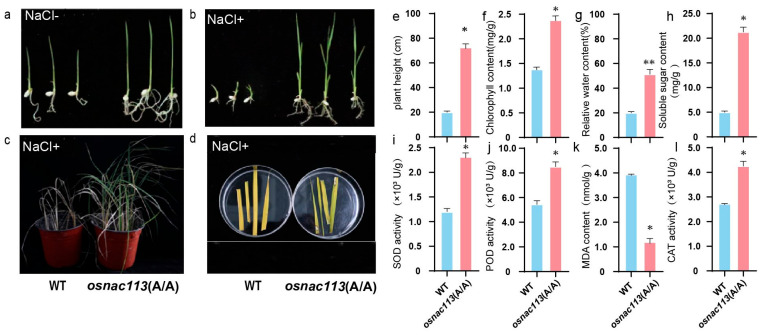
Phenotypic characteristics of osnac113 mutants and determination of physiological indices after salt stress. (**a**) Phenotypic characteristics of osnac113 mutants under normal conditions. (**b**) Phenotypic characteristics of osnac113 mutants under salt stress conditions. (**c**) Twelve-week-old WT and mutants were subjected to 14 days of salt stress. (**d**) Leaf changes in wild-type and mutant following salt stress. (**e**) Plant height. (**f**) Chlorophyll content. (**g**) Relative water content. (**h**) Soluble sugar content. (**i**) SOD activity. (**j**) POD activity. (**k**) MDA content. (**l**) CAT activity. All biochemical calculations performed on DW. Bars represent the mean ± SE of three independent experiments. * and ** represent significant differences at *p* < 0.05 and *p* < 0.01 compared to WT.

The mutants showed better growth under normal growth conditions (Figure 3a). One week after the *OsNAC113* mutant and wild-type rice were treated with 200 mM NaCl, the mutant plants showed increased resistance (Figure 3b). The rice seedlings were photographed and observed after treatment with 200 mM NaCl (Figure 3c). The mutant plants exhibited higher resistance to salt stress. The leaves of mature wild-type and mutant plants were salted (200 mmol/L, 3 d) and photographed for observation. The survival rate of the mutant was significantly higher than that of the wild type (Figure 3d). In summary, the salt resistance of *osnac113* was enhanced during germination and maturation. The plant height of mutants was increased two to three times compared to wild-type under stress conditions (Figure 3e). The chlorophyll content in the mutant was 54% higher than that in the wild type, indicating that the mutant had stronger resistance to salt stress (Figure 3f). The relative water content of wild-type plants and plants subjected to salt stress (200 mM) for 7 d was determined. The water content of the OsNAC113 mutant is 3 to 4.5 times higher than that of the wild type (Figure 3g). Additionally, the soluble sugar content is 33% greater than that of the wild type (Figure 3h). At this time, the ROS clearance mechanism in the cell was activated, and ROS-related enzymes included catalase (CAT), peroxidase (POD), and superoxide dismutase (SOD). The activities of SOD-, POD-, and CAT-related enzymes in *OsNAC113* were 18.7%, 24.5%, and 22.5% higher, respectively, than those in the wild type. The experimental results showed that the MDA (Malondialdehyde) content in the *OsNAC113* mutant was 6% lower than that in the wild type (Figure 3i–l). This indicates that the oxidative damage to the *OsNAC113* mutant was less under salt stress.

### 2.4. RNA-Seq Analysis of Mutants and Wild-Type Under High-Salt Stress Conditions

**Figure 4:**
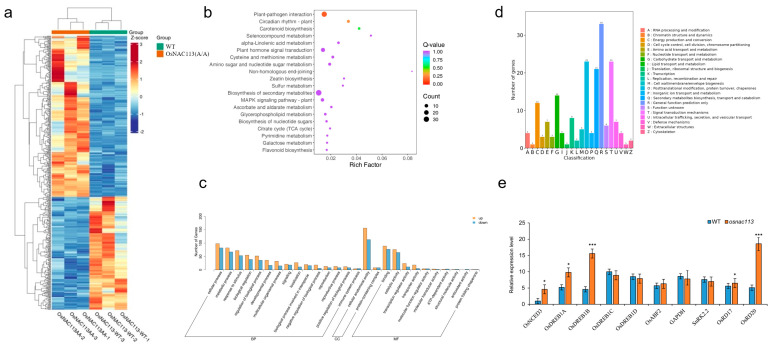
Knockout of OsNAC113 induces genome-wide changes in plants. (**a**) WT and osnac113 genome-wide expression analysis (drought−/+). (**b**) KEGG analysis targeting DEG shared by WT vs. osnac113 (T/T) vs. osnac113 (−1/−1): drought and WT vs. osnac113 (T/T) vs. osnac113 (−1/−1): drought. (**c**) Gene ontology (GO) classification of DEG shared by WT vs. osnac113 (T/T) vs. osnac113 (−1/−1): drought and WT vs. osnac113 (T/T) vs. osnac113 (−1/−1): drought+. (**d**) Classification of the signaling pathways of DEG. (**e**) Screen and measure the expression levels of 10 significantly differentially expressed genes. The relative expression level of *OsNAC113* was measured by RT-qPCR at the indicated times. Error bars indicate SE based on three independent biological replicates. * and *** represent significant differences at *p* < 0.05 and *p* < 0.001 compared to WT.

To further reveal the mechanism by which *OsNAC113* enhances salt tolerance in rice at the whole-genome level, RNA-seq analysis was performed on WT and *OsNAC113* mutants at 9 weeks of normal growth, and on WT and *OsNAC113* mutants at 8 weeks of normal growth and 1 week of salt stress. A total of 468 differentially expressed genes between *OsNAC113* (A/A) and WT plants showed decreased expression (Figure 4a). The KEGG enrichment analysis of DEG showed that the top three enrichment pathways with the greatest differences were “secondary metabolite biosynthesis”, “plant hormone signaling pathway”, and “MAPK signaling pathway”, accounting for 30.63%, 13.51%, and 8.11%, respectively. In addition, pathways such as “carotenoid biosynthesis,” “alpha linolenic acid metabolism,” “zeaxanthin biosynthesis,” and “flavonoid biosynthesis,” as well as “ascorbic acid and aldose metabolism,” were significantly enriched (Figure 4b). GO annotation analysis confirmed the effect of the *OsNAC113* knockout on the biological processes involved. The results showed that 27 GO classifications were annotated by these differentially expressed genes, including 2 belonging to the cellular component, 10 belonging to the molecular function, and 15 belonging to the biological process. In the cellular component, the protein-containing complex undergoes a change. In the molecular function signaling pathway, modifications related to binding and catalytic activity occurred, indicating that OsNAC113 may affect the synthesis of organic compounds. Biological processes, such as cellular processes, metabolic processes, and response to stimuli, were all affected, suggesting that the knockout of OsNAC113 may alter the growth cycle of the mutant (Figure 4c).

Among the differentially expressed genes, 12 were related to energy production and conversion, 7 to amino acid transport and metabolism, 14 to carbohydrate transport and metabolism, 4 to lipid transport and metabolism, 2 to replication, recombination, and repair, and 5 to cell wall, membrane, and envelope biogenesis, along with post-translational modification, protein turnover, and chaperones. Additionally, there were 23 genes involved, including 21 related to secondary metabolite biosynthesis, transport, and catabolism, 23 related to signal transduction mechanisms, 7 related to intracellular transport, secretion, and vesicle transport, and 4 related to defense mechanisms (Figure 4d). These results suggest that the response of *OsNAC113* to salt stress may be mediated through the aforementioned biological processes.

We measured the expression levels of 10 significantly differentially expressed genes and observed that the transcriptome sequencing results were consistent with the expression trends of these genes, indicating that the relative expression levels of the genes obtained by transcriptome sequencing were reliable. The expression levels of *OsNCED3*, *OsDREB1A*, *OsDREB1B*, and *OsRD20* significantly increased in mutant plants, which were 5.1, 1.9, 4.2, and 4.8 times higher than those in wild-type plants. The rate-limiting enzyme for the biosynthesis of ABA in higher plants was 9-cis-cyclocarotenoid dioxygenase (NCED) (Figure 4e).

### 2.5. Differential Signaling Pathways and Metabolite Changes Caused by Changes in Gene Expression

**Figure 5:**
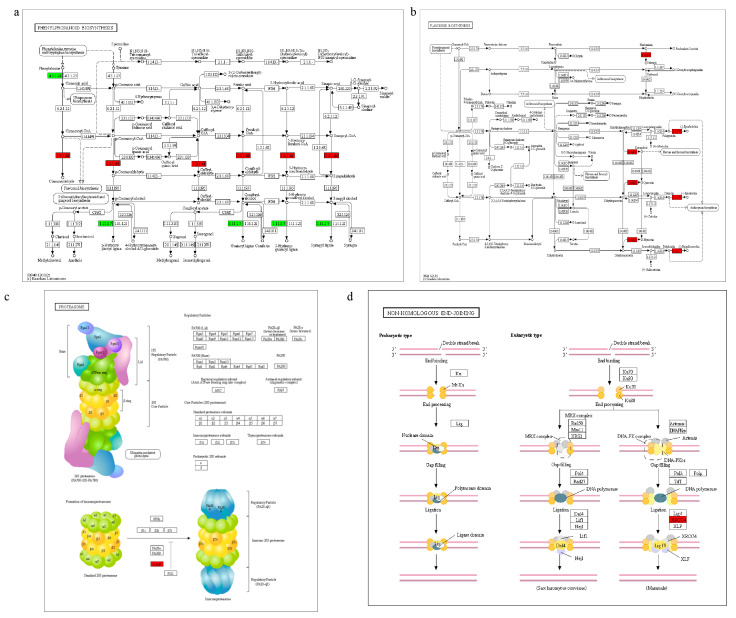
Knockout of OsNAC113 results in signal pathway changes. (**a**) Phenylpropanoid and (**b**) flavonoid biosynthesis. (**c**) Proteasome; (**d**) non-homologous end-joining. The biosynthetic schemes were taken from the KEGG library. Red represents upregulated expression, green represents downregulated expression.

In the phenylpropanoid biosynthesis pathway, cinnamoyl-CoA reductase [EC: 1.2.1.44] was upregulated, leading to an increase in the expression of cinnamaldehyde. In the mutant, increased cinnamaldehyde levels improved stress resistance and enabled the mutant to effectively adapt to high-salt stress (Figure 5a). Flavonol synthase [EC: 1.14.20.6] was upregulated in the flavonoid biosynthesis pathway. Flavonoids are the most widely distributed class of plant compounds. Flavonols protect the mutants from various adverse stimuli. Anthocyanin reductase [EC: 1.3.1.77] was also upregulated (Figure 5b). Anthocyanins play a protective role in these mutants. They can absorb ultraviolet radiation and act as antioxidants, preventing damage to photosynthetic pigments and other cellular structures and reducing the degree of damage. Flavonoids and anthocyanins can effectively protect mutants from damage and improve their stress resistance. During synthesis of the mutant proteasome, the expression of the POMP gene is upregulated, and its product is the mature protein of the proteasome. The primary function of proteasomes is to degrade unwanted or damaged proteins in cells, which is beneficial for plant damage repair under stress, leading to the increased resistance of mutants to salt stress (Figure 5c). In the pathway of synthesizing non-homologous end connections, the expression level of the DNA repair protein XRCC4 is upregulated. When the body grows rapidly or is inhibited by stress, exogenous nucleotides must be added to ensure tissue growth and normal function (Figure 5d).

**Figure 6:**
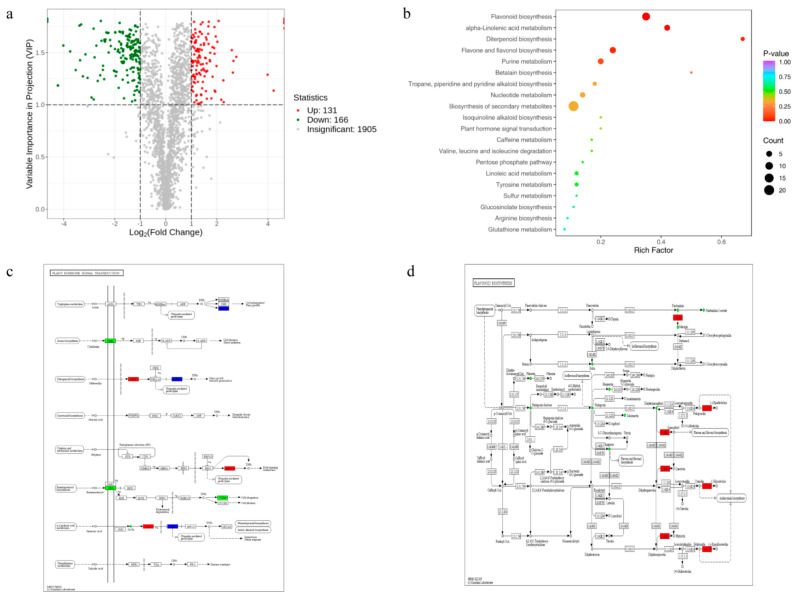
Knocking out OsNAC113 leads to changes in plant metabolism. (**a**) Volcanic diagram of differential metabolite quantity. (**b**) KEGG enrichment of differential metabolite pathways results. (**c**) Plant hormone signal transduction. (**d**) Flavonoid biosynthesis. Red represents upregulated expression, green represents downregulated expression.

Six samples (each three biological/technical replicates from mutant line and WT), divided into *OsNAC113* (+A/+A) and WT, were selected for metabolic research. Comparative analysis of the differentially expressed metabolites of OsNAC113 revealed that after OsNAC113 was knocked out, the expression of metabolites in rice plants changed. The number of differentially expressed metabolites in the samples was counted, and 1905 metabolites were detected. Of these, 297 metabolites were differentially expressed. A total of 131 metabolites exhibited increased expression, and 166 metabolites exhibited decreased expression (Figure 6a). In the scatter plot of mutant differential metabolites, the top three enriched pathways are “secondary metabolite biosynthesis,” “metabolic pathway,” and “flavonoid biosynthesis. Simultaneously, pathways such as “biosynthesis of flavonoids and flavonols,” “unsaturated fatty acid metabolites,” “purine metabolism,” and “nucleotide metabolism,” as well as ABC transporters and plant hormone signaling pathways, were significantly enriched (Figure 6b).

After knocking out OsNAC113, the upregulation of SAUR leads to an increase in the accumulation of tryptophan, which affects the hormone regulation of the entire plant as a prerequisite for auxin synthesis. The gene Gibberellin Insensitive Dwarf1 (*GID1*) is upregulated during molecular biosynthesis, and the *GID1* receptor protein is a key factor in the gibberellin signaling pathway. Upregulation of *ERF1/2* expression has been observed in cysteine and methionine metabolism. During the biosynthesis of brassinosteroid (BR), *BRI1* and *TCH4* are downregulated, suggesting that BR inhibits the transcription of synthetic genes through a negative feedback mechanism. *COl1* has been upregulated in the jasmonic acid pathway system, leading to the accumulation of downregulated expression of *JAR1* enzyme, ultimately resulting in changes in the entire synthesis system of JA (Figure 6c).

In the flavonoid biosynthesis pathway, the expression of flavonol synthase (EC1.14.20.6) is upregulated (Figure 6d). The reaction formula is as follows: dihydroflavonol + 2-oxoglutaric acid + O_2_ = flavonol + succinate + CO_2_ + H_2_O. Dihydroflavonol, 2-oxoglutaric acid, flavonol, and succinate upregulated expression is observed in all synthetic pathways. Ultimately, it leads to the upregulation of ornithine decarboxylase (EC: 4.1.1.17) in arginine and pro metabolic pathways, thereby affecting cell proliferation, growth, and differentiation. Changes in the expression levels of substances in these pathways in the mutants enhanced their stress resistance (Supplementary Figure S4). Proteins in living organisms rely on complex networks comprising multiple protein interactions. The STRING Protein Interaction Database (http://string-db.org, accessed on 22 July 2024) was used to analyze differential gene–protein interaction networks to better understand the functions and mechanisms of the proteins (Supplementary Table S3). Table S3 presents 24 differentially expressed genes corresponding to their protein interaction scores in the database. The protein connectivity of differentially expressed genes was relatively high, indicating that they play a key role in maintaining system balance and stability and are closely related to plant stress resistance.

## 3. Discussion

CRISPR/Cas gene editing technology has recently enabled the rapid and accurate production of new germplasms within a short time period, which is of considerable importance for investigating rice genome function, expression regulation, and genetic improvement of germplasm resources. Hui et al. (2022) created a new allele of betaine aldehyde dehydrogenase 2 (*OsBADH2*) and cultivated a three-line hybrid rice variety with enhanced grain aroma [11], while Zhu et al. developed an efficient multi-gene vector system, Trans Gene Stacking II (TGSII), using CRISPR/Cas technology, which enables targeted synthesis of anthocyanins in the endosperm. Eight key genes related to anthocyanin synthesis have been transferred to rice receptors, creating a new germplasm, “Purple Crystal Rice,” rich in anthocyanins [12]. Zhao et al. knocked out the rice histone demethylase *JMJ710* and observed that the JMJ710 gene, which regulates its synthesis, was a negative regulator of drought stress response genes, leading to strong drought resistance in rice mutants [13]. Zu et al. isolated the inhibitor sop10 of the pseudouridine synthase gene *OsPUS1-1* in indica rice, and observed that mutants without sop10 function exhibited reduced levels of ROS, increased tolerance to low temperature stress, and maintained yield [14].

NAC TFs are involved in the regulation of plant responses to various stressors, including salinity, cold, and drought [15,16]. The effects of salt stress on plants are mainly manifested in three aspects: (1) high salt content results in osmotic stress, thereby preventing plants from absorbing water; (2) plants absorb toxic Na^+^ and Cl^−^ ions, disrupting the body’s ion balance; and (3) salt stress can inhibit plant growth and promote aging by disrupting redox homeostasis [17]. The results of this study indicated that the *osnac113* mutant tends to be induced by salt stress. *OsNAC113* was specifically induced under salt stress conditions, but showed no significant response to IAA and hydrogen peroxide stress. Under normal conditions, *OsNAC113* is mainly expressed in the leaves and leaf sheaths, with a relative expression level as high as 40–50 times that in the roots. In addition, we observed that the expression level of OsNAC113 increased within 1–6 h of salt treatment and reached its peak at 3 h after treatment. These results indicate that *OsNAC113* expression is tissue-specific and affects salt stress tolerance in rice.

In rice, *OsNCED3* promotes ABA synthesis and enhances abiotic stress tolerance [18]. In higher plants, NCEDs are key enzymes that control ABA biosynthesis and belong to a differentially expressed gene family. The transcription factors DREBs/CBFs specifically interact with the dehydration response element/C-repeat (DRE/CRT) cis-acting element (nucleoid motif: G/ACCGAC). Overexpression of OsDREB1A in Arabidopsis can induce overexpression of the DREB1A gene, thereby enhancing Arabidopsis tolerance to various stresses, such as high salt [19]. This indicates that OsDREB1A and DREB1A have similar functions; OsWRKY28 enhances salt tolerance in rice by directly binding to the OsDREB1B promoter and increasing its transcriptional activity, while negatively regulating ABA-mediated rice seedling establishment [20]. RD20 is a stress-induced Arabidopsis gene belonging to the caleosin family. Compared to the wild type, rd20 knockout plants exhibited higher transpiration rates, which were related to increased stomatal opening and reduced drought tolerance. These results support the role of RD20 in drought tolerance under water-deficient conditions via stomatal control [21]. The expression levels of *OsNCED3*, *OsDREB1A*, *OsDREB1B*, and *OsRD20* significantly increased in the mutant, and the expression of these genes was closely related to the response of plants to abiotic stress. Therefore, the mutants may enhance their tolerance to salt stress by regulating the pathways in which they participate.

Excessive production of ROS is a fundamental plant response to salinity. Under salt stress, plant photosynthesis is inhibited, and the ROS produced during photosynthesis directly or indirectly affect the decomposition of chlorophyll and electron transfer. Plant chloroplasts and mitochondria are the prominent organelles producing ROS in cells and mediating the induction of pressure tolerance. The regulation of the antioxidant system includes both enzymatic and non-enzymatic components [22]. In salt-tolerant rice lines, OsP5CS expression is upregulated, enhancing the accumulation of the key osmotic protective molecule pro. Plants can protect their cells from ROS damage and improve salt tolerance in rice [23]. *OsGPX2*, *OsGPX4*, and *OsGPX5* in the rice glutathione peroxidase (GPXs) gene family are associated with the AsA GSH pathway and contribute to improved salt tolerance in rice [24]. Overexpression of the glutathione S-transferase gene *OsGST4* can clear ROS and increase salt tolerance [25]. In this study, the *OsNAC113* mutant showed enhanced tolerance to salt stress. Under salt stress, the *OsNAC113* mutant showed an increase in chlorophyll content; enhanced activities of SOD, POD, and CAT; a decrease in MDA content; and an increase in soluble sugar content. The *OsNAC113* mutant exhibited increased resistance to salt stress, indicating that the *OsNAC113* transcription factor may regulate genes related to peroxidases in the antioxidant system.

Plant hormones secreted by complex mechanisms are key factors affecting salt tolerance in rice and play a crucial role in plant adaptation to various stresses. Plant hormones are endogenous compounds that act as plant growth regulators at their synthesis sites, or when transported to other sites under different environmental and stress conditions. Abscisic acid is a stress-responsive plant hormone that facilitates the adaptation of plants to adverse environmental conditions by inhibiting seed germination and seedling growth. ABA plays an important regulatory role in salt stress processes. Jasmonic acid is involved in regulating plant life processes in response to various forms of abiotic stress, potentially contributing to enhanced resistance [26]. The expression of the gene OsEIN2 in the ethylene signaling pathway stimulates salt tolerance in rice [27]. In this study, *OsNAC113* responded to adversity stress, and ABA and GA_3_ treatments induced the expression of *OsNAC113*. During tryptophan metabolism, SAUR expression is upregulated, resulting in increased tryptophan production. Higher plants contain a substance that is structurally similar to IAA, and its precursor is tryptophan. The gene JAZ expression is upregulated, and JA is a lipid-derived natural plant hormone that can induce stomatal closure, which is beneficial for maintaining water in plants. The gene GID1 is upregulated during molecular biosynthesis. Gibberellin (GA) is a hormone that plays a crucial role in plant growth and regulates it under abiotic stress. In this study, we constructed a knockout mutant of *OsNAC113* and observed that *OsNAC113* was involved in regulating multiple regulatory pathways at the molecular metabolic level, thereby enhancing plant salt tolerance.

ABC transporters in plants are involved in various functions such as secondary metabolite transport [28], heavy metal detoxification, antibiotic transport [29], and plant hormone transport [30]. Therefore, the functional annotation and analysis of differential metabolites observed in *OsNAC113* gene knockout plants indicate that changes in metabolites representing ABC transporters also affect the production of ABC transporters, thereby regulating the growth and development of rice. MAPKs are relatively conserved eukaryotic protein kinases, and they are involved in regulating signal transduction under stress and adversity [31]. Salt stress leads to the activation of MPK6, which can phosphorylate the C-terminus of SOS1, thereby enhancing salt tolerance in Arabidopsis [32]. MAPKs are involved in the response and adaptation of plants to stress, thereby regulating the homeostasis of intracellular ROS [33]. Notable differences were observed in the gene expression levels of the ABC transporter and MAPK signaling pathways in the *OsNAC113* mutant, revealing the molecular mechanisms underlying the response regulation of *OsNAC113* under high-salt stress conditions.

Flavonoids play a crucial role in enabling plants to resist biotic or abiotic stresses and are beneficial for plants in resisting external stress [34]. The Arabidopsis R2R3-MYB transcription factor PFG3 enhances plant tolerance to drought and osmotic stress by regulating flavonoid biosynthesis. In rice, the MYB-bHLH-WD40 protein complex synergistically regulates anthocyanin biosynthesis-related genes [35]. The important structural genes OsPAL, OsC4H, and Os4CL, which are involved in anthocyanin biosynthesis, mainly play a role in lignin synthesis and stress resistance [36]. In this study, flavonol synthase [EC: 1.14.20.6] was upregulated. Flavonoids are the most widely distributed class of compounds in plants. Flavonols protect plants from various environmental stimuli. The expression of anthocyanin reductase [EC: 1.3.1.77] was also upregulated. Anthocyanins play protective roles in plants. They can absorb ultraviolet radiation and act as antioxidants, preventing damage to photosynthetic pigments and other cellular structures and reducing the degree of damage. In summary, *OsNAC113* may affect the above pathways and respond to salt stress.

## 4. Materials and Methods

### 4.1. Plant Materials and Cultivation Conditions

All rice materials used in this experiment, including wild-type Japanese clear rice (Nipponbare, *Oryza sativa* L. Japan) and *osnac113* mutant plants, were grown in a light incubator at 28 °C for 16 h light (3000 Lux)/8 h dark conditions. The substrate used for tissue culture is MS medium. Wild-type and mutant plants with consistent growth are subjected to salt stress treatment simultaneously, and the above plant material was subjected to stress treatment with 200 mM saline solution one month after the growth of T1 seeds. The material growth environment is in a 3000 lux light incubator, and the photoperiod is 16 h light and 8 h darkness. After performing normal germination and growing in the soil for 4 weeks, different abiotic stresses were applied to rice to detect the response of *OsNAC113* to different stimuli. Like low temperature stress of 4 °C, high temperature stress of 42 °C, simulated drought stress of PEG6000 (20%, *w*/*v*), high salt NaCl (200 mM), 1% H_2_O_2_ stress, 100 mM IAA hormone induction, 100 mM ABA hormone induction, and 100 mM GA_3_ induction. Plant RNA was extracted and transformed into cDNA for RT-qPCR analysis at 0, 1, 3, 6, 9, and 12 h. Each sample had three biological replicates.

### 4.2. Real-Time Quantitative PCR System and Conditions

The reaction solution was prepared using TB Green Premix Ex Taq II (R820A) from BioNTech (Mainz, Germany). The reaction mixture (20 μL) comprised the following components: total RNA reverse transcribed into cDNA (kit: PrimeScript™ RT reagent Kit, RR037Q), 1 μL cDNA, 0.5 μL upstream primer, 0.5 μL downstream primer, 10 μL 2× SYBR Green Mix, and 8 μL H_2_O. A qPCR reaction was performed on the My IQ quantitative PCR instrument of the BIO-RAD company (Hercules, CA, USA). The quantitative data were analyzed using BIO-RAD software (V2.0), and the relative expression values of genes were calculated using the ΔΔCt analysis method. The qPCR reaction is as follows: 94 °C pre-denaturation, 30 s, 1 cycle; denaturation at 94 °C, 5 s, 40 cycles; annealing at 58 °C. 15 s, 40 cycles; and extend at 72 °C for 40 cycles. The verification of the specificity of amplification was performed by melting curves (65 °C to 95 °C, increments of 0.5 °C). The ΔΔCt analysis method is used to calculate the relative expression values of genes for data analysis and calculation.

### 4.3. Subcellular Localization Requires the Construction of a Target Protein Expression Vector

In order to confirm the location of *OsNAC113*, the pBWA (V) HS-OsNAC113-Glosgfp10509 vector was constructed and incorporated into rice protoplasts. The plasmid encodes OsNAC113 fused to green fluorescent protein (GFP), and the empty GFP vector NLS::eGFP served as a control. Seedlings of rice were grown in the dark at 25 °C in a growth room for 1–2 weeks before isolating protoplasts. Healthy fresh rice seedlings were cut into fine segments and digested with an enzyme solution (1.5% cellulase R10, 0.75% Macerozyme R10, 0.6 M mannitol, 10 mM MES pH 5.7, 10 Mm CaCl_2_, and 0.1% BSA). After 3 h digestion with gentle shaking (20–30 rpm), protoplasts were isolated by filtration through 40 µm nylon meshes into round-bottom tubes. Pellets were collected at 80 g for 3 min and washed with W5 solution (154 mM NaCl, 125 mM CaCl2, 5 Mm KCl, and 2 mM MES pH 5.7), and the protoplasts were kept on ice for 30 min. They were then resuspended in MMG solution (0.4 M mannitol, 15 mM MgCl_2_, and 4 mM MES pH 5.7). Protoplast transformation was carried out in PEG solution (40% (*w*/*v*) PEG 4000, 2 M mannitol, and 0.1 CaCl_2_. Transformation mixtures (10 µg of target plasmid or, additionally, add 10 ug of marker plasmid for cotransformation with 200 µL protoplasts in 250 µL of PEG solution) were agitated gently. After 30 min at room temperature in the dark, protoplasts were harvested and washed with 800 ul of W5 solution. They were then centrifuged and resuspended in 2 mL W5 solution and cultured in the dark at room temperature, generally for 16–24 h. Then, the plasmid was viewed under a Laser scanning confocal microscope. The promoter used for the carrier is the *35S* promoter. The eukaryotic resistance of the carrier is hygromycin, and the prokaryotic resistance is kanamycin. The microscope used was a Nikon C2-ER Laser Confocal Microscope, and the manufacturer is Nikon. After 24 h of cultivation, the proteins were separated and purified, and protein gel electrophoresis was performed to observe whether the green fluorescent protein was fused with osnac113. Finally, the positional distribution of green fluorescent protein was detected using a fluorescence microscope.

### 4.4. Construction of Rice sgRNA

Based on the recognition and cleavage rules of CRISPR/Cas9 for target sites, the design of sgRNA in the *OsNAC113* (https://planttfdb.gao-lab.org/tf.php?sp=Osj&did=LOC_Os08g10080.1, accessed on 11 June 2023) gene coding region was conducted using the database http://CRISPR.hzau.edu.cn/CRISPR (accessed on 11 June 2023). The upstream and downstream primers of each target site were diluted to a concentration of 100 μmol. Additionally, 10 μL of each was collected, denatured for 5 min at 98 °C, cooled naturally, and the annealing product was diluted 20 times for later use. BsaI was used to perform single-enzyme digestion on pZHY988 [37], and the restriction endonuclease instructions of New England Biolabs (NEB) were used to establish the digestion system and digestion conditions. Subsequently, NEB’s T4 ligase was used for ligation. The knockout site of the gene was selected at the position of the second exon (Figure 2a), and the corresponding sgRNA was designed at this position.

### 4.5. Rice Knockout Vector Transformation

Agrobacterium-mediated transformation was used to infect and transform rice callus tissues. After infection, the callus was co-cultured in the dark for three days and then washed five times with sterile water. The callus was washed once with sterile water containing 500 mg/L carbenicillin (Carb) and transferred to a screened solid culture containing 500 mg/L Carb + 50 mg/L Hyg medium for 2 months. In the later stage, regenerated seedlings were induced to root in regeneration medium [38].

### 4.6. Identification of OsNAC113 Knockout Mutant in Rice and Physiological Measurements

After 3 months of transformation, DNA was extracted from regenerated plants, and PCR product sequencing was used to identify the regenerated seedlings. After obtaining regenerated seedlings, individual plant genomes were extracted, and Sanger sequencing (Genewiz, Suzhou, China) was used to identify the genotype after gene knockout. The sequencing results were compared with the wild type to obtain the knockout genotype. Sequencing results were analyzed to identify the different knockout types of *OsNAC113*. Relative water content (RWC) testing: take a portion of the leaves and weigh the weight as fresh weight (FW); soak the leaf culture dish in water overnight; wipe off the moisture on the surface of the leaves and call the mass swelling (TW); 45 °C, 24 h, fully dry the leaves, and then weigh the mass as dry weight (DW). The formula for calculating relative moisture content is RWC = (FW-DW)/(TW-DW).

The method for determining chlorophyll content is as follows: Take 1 g of fresh leaves and grind them into powder with liquid nitrogen. Transfer all the powder to a 15 mL centrifuge tube, place it on ice, add 5 mL of 80% acetone for 30 min, and shake thoroughly three times during this period. At 4 °C, 10,000 rpm, centrifuge for 10 min, take the supernatant, and dilute it to 5 mL with 80% acetone. Use 80% acetone as a blank control to determine the OD values of the samples (OD645 and OD663). The formula for calculating chlorophyll content is C (a + b) (mg/L) = OD663 × 8.02 + OD645 × 20.2.

The method for the determination of superoxide dismutase (SOD) activity is as follows: Take leaves from the same location of wild-type and mutant plants. If the treatment conditions require simultaneous processing, take samples and grind 5 mL of pre-cooled 0.05 mol/L PBS (pH 7.8) on ice until homogenized, then transfer to a centrifuge tube; centrifuge at 4 °C, 10,000 rpm for 20 min, then collect the supernatant and store at 4 °C. Prepare reaction mixture: 40.5 mL 14.5 mmol/L Met solution + 1.5 mL 3 mmol/L EDTA-Na2 solution + 1.5 mL 2.25 mmol/L NBT solution + 1.5 mL 60 µ mol/L riboflavin solution. Take 3 mL of the reaction mixture and 40 µL of the enzyme solution separately and place them in test tubes as sample tubes. Simultaneously perform two control tubes: control—3 mL reaction mixture + 40 µL PBS to measure the maximum photoreduction value, and initial control—set to zero when measuring 3 mL reaction mixture + 40 µL PBS (tin foil paper in the dark). The sample tube and control tube undergo a 4000 Lx light reaction at 25 °C for 20 min. The initial control was zeroed, and the absorbance value OD560 was measured sequentially for the samples. The calculation formula is SOD activity (U/g FW) = [(ACK-AE) × Vt]/(half ACK × W × VS), where ACK is the absorbance value of the control tube, AE is the absorbance value of the sample tube, Vt is the total volume of enzyme solution, W is the fresh weight of the sample, and vs. is the amount of enzyme solution used during measurement.

The method for the determination of peroxidase (POD) activity is as follows: weigh an appropriate amount of leaves from the same part of different strains (subjected to stress treatment for the same time) and place them in a mortar. Add 5 mL of pre-cooled 0.05 mol/L PBS (pH 7.8), grind them thoroughly on ice until homogenized, and then pour them into a 10 mL centrifuge tube; centrifuge at 4 °C and 12,000 rpm for 20 min, then collect the supernatant and store at 4 °C; preparation of reaction mixture: 50 mL of 0.1 mol/L PBS (pH 6.0) + 28 µL of guaiacol (2-methoxyphenol) are heated and stirred, cooled, and then mixed with 19 µL of 30% H_2_O_2_ and stored at 4 °C; take 3 mL of reaction mixture and 40 µL of enzyme solution separately and place them in test tubes as sample tubes. An amount of 3 mL reaction mixture + 40 µL PBS control zeroing; measure the change in OD470 at 40 s for 3 mL reaction mixture + 40 µL PBS. The formula for calculating POD activity is (U/g FW) = (△ OD470 × Vt)/(W × VS × 0.01 × T), where △ OD470 is the absorbance change within 40 s, Vt is the total volume of enzyme solution used, W is the fresh weight of the sample, VS is the amount of enzyme solution used during measurement, and T is the reaction time.

The method for catalase (CAT) activity assay is as follows: weigh leaves of different strains and parts of the same mass (under identical stress conditions and time), and place them in a mortar. Grind 5 mL of pre-cooled 0.05 mol/L PBS (pH 7.8) on ice until homogenized, then pour into a centrifuge tube for storage. At 4 °C, 10,000 rpm, centrifuge for 20 min and take the supernatant, which is the crude enzyme extract. Store the crude enzyme extract at 4 °C. Reaction mixture: Mix 100 mL of 0.1 mol/L PBS (pH 7.0) and 0.1546 mL of 30% H_2_O_2_ evenly. PBS was used as a control to zero, and the changes in OD240 of the 2.9 mL reaction mixture and the 0.1 mL enzyme solution were measured within 40 s. The calculation formula for CAT activity (U/g FW) is (△ OD240 × Vt)/(W × VS × 0.01 × T). Among them, △ OD240 is the absorbance change within the reaction time, Vt is the total volume of enzyme solution, W is the fresh weight of the sample, VS is the amount of enzyme solution used during the measurement, and T is the reaction time [38].

Determination of soluble sugar content by anthrone colorimetric method: soluble sugars generate furfural under the action of sulfuric acid, which then undergoes dehydration and condensation reactions with anthrone to form a green complex. The color intensity and sugar content are linearly related. Measure the absorbance value of soluble sugars using a spectrophotometer with a wavelength of 625 nm. Based on the standard curve, the soluble sugar content in the sample can be calculated. It should be noted that the color presented by the reaction between anthrone reagent and sugar changes over time. In order to accurately measure, it is necessary to complete the measurement within the specified time and record the data.

For phenotypic analysis of seedlings, WT and *OsNAC113* mutant seeds were grown to the 4-week-old seedling stage in soil in pots, then subjected to salt stress. After 7 days of treatment, physiological measurements were carried out as described in our previous study [39].

### 4.7. Off-Target Efficiency Detection

Using the Cas9 OFF-finder (www.rgenome.net/cas-offinder/, accessed on 11 December 2024) website for prediction, we selected the 10 most likely off-target positions for detection, searched for their corresponding site sequences, designed primers based on the search sequences, and performed sequencing analysis of the amplified PCR products to verify whether there were off-target conditions.

### 4.8. Omics Sequencing and Data Analysis

RNA-seq and Metabolome sequencing were performed by Wuhan Maiweier Biotechnology Co., Ltd. (Wuhan, China) The transcriptome sequencing instrument system is Illumina NovaSeq 6000 (Thermo Fisher Scientific, Waltham, MA, USA). The liquid phase system used for data collection is Thermo’s ultra-high-pressure liquid phase system, UltiMate 3000 UPLC. The chromatographic column model and specification used is ACQUITY UPLC T3 (100 mm × 2.1 mm, 1.8 µm, Waters, Wilmslow, UK). The high-resolution mass spectrometer used for collection is a TripleTOF 6600 (SCIEX, Framingham, MA, USA) time-of-flight mass spectrometer. Wild-type and *OsNAC113*(+*A*/+*A*) with consistent growth were subjected to salt treatment (200 mM) for seven days before sampling. Transcriptome sequencing included total RNA extraction, mRNA enrichment, double-stranded cDNA synthesis, end-effector repair, fragment selection, PCR amplification, and library detection. The experimental process was as follows: after the library was constructed, the RNA sample concentration was ≥250 ng μL^−1^, pure OD260/280 = 1.8–2.2, OD260/230 ≥ 2.0, and the quality RIN > 7.0. By using fastp and Trimomatic to perform quality control on the raw data, high-quality quality-control data can be obtained. Using Hisat software (V1.0) to compare quality control data to the reference genome, assembling and estimating transcript abundance using StringTie software (V1.0), and predicting differential expression of genes and transcripts using DESeq2 software (V1.0). Perform GO and KEGG enrichment analysis of differentially expressed genes using the clusterProfiler package in R language.

Collect and process samples. Conventional samples (cells, etc.) are washed with PBS and centrifuged at 4 °C to obtain a precipitate. Precipitation of proteins and extraction of metabolites. QC (Quality Control) sample preparation: Take 10 µL of each sample and mix it to form a QC sample. Metabolomics studies were conducted using high-throughput mass spectrometers such as SCIEX 6500+.

### 4.9. Bioinformatics Analysis

The NCBI database was used to query the upstream 2 Kb sequence of the *OsNAC113* start codon (ATG) as the promoter sequence, and EXPASy, Netphos3.1, TMHMM2.0, ProtScale (V1.0), and pfam were used to predict the gene characteristics and secondary structure of *OsNAC113* (Supplementary Table S2).

### 4.10. Statistical Analysis

Based on transcriptome data, RESM software was used to perform background correction and standardization on gene expression data. Filter genes with low expression levels and low coefficient of variation. Using R software (R version 4.1) and WGCNAWeighted. Gene Correlation Analysis (WGCNA) was performed using the R version 1.6.6 package and the Pearson correlation coefficient. Soft Threshold power β estimates e-value to fit the network into a scale-free topology, and the topology of each gene pair overlapping measurement (TOM) is used to construct dissimilarity matrices and to perform hierarchical clustering with arithmetic mean (UPGMA). Unweighted grouping method for classes: The best clustering scheme is implemented using the Dynamic Tree Cut R package to screen genes co-expressed with *OsNAC113*.

LC-MS/MS analyses were performed using a UHPLC system (Vanquish, Thermo Fisher Scientific) with a Waters ACQUITY UPLC BEH Amide (2.1 mm × 50 mm, 1.7 μm) coupled to an Orbitrap Exploris 120 mass spectrometer (Orbitrap MS, Thermo). The mobile phase consisted of 25 mmol/L ammonium acetate and 25 mmol/L ammonia hydroxide in water (pH = 9.75) (A) and acetonitrile (B). The auto-sampler temperature was 4 °C, and the injection volume was 2 μL. The Orbitrap Exploris 120 mass spectrometer was used for its ability to acquire MS/MS spectra on information-dependent acquisition (IDA) mode in the control of the acquisition software (Xcalibur, Thermo). In this mode, the acquisition software continuously evaluates the full scan MS spectrum. The ESI source conditions were set as follows: sheath gas flow rate as 50 Arb, Aux gas flow rate as 15 Arb, capillary temperature 320 °C, full MS resolution as 60,000, MS/MS resolution as 15,000, collision energy SNCE 20/30/40, and spray voltage as 3.8 kV (positive) or −3.4 kV (negative), respectively. The raw data were converted to the mzXML format using ProteoWizard and processed with an in-house program, which was developed using R and based on raw data, were converted to the mzXML format using ProteoWizard and processed with an in-house program, which was developed using R and based on XCMS, for peak detection, extraction, alignment, and integration. Then, an in-house MS2 database was applied in metabolite annotation. The cutoff for annotation was set at 0.3

## 5. Conclusions

The expression level of *OsNAC113* is highest in the leaves during both the seedling and mature stages. Its expression changes indicate that it responds to various adverse biological stresses. Through genetic transformation of rice, knocked-out *OsNAC113*-regenerated plants were obtained. After Sanger sequencing identification and analysis, mutant *OsNAC113* (−1/−1) and *OsNAC113* (+A/+A) were obtained, providing a material basis for subsequent experiments. Under salt stress conditions during germination and seedling stages, knocking out *OsNAC113* enhances plant salt tolerance. Physiological indicators testing showed that the relative water content, chlorophyll content, and oxidoreductase-related activity of the mutant were higher than those of the wild type, while the accumulation of MDA and other harmful substances was higher in the wild type. Analyze the whole genome expression changes in wild-type and mutant under stress based on RNA-seq analysis. After stress, significantly differentially expressed genes were screened through bioinformatics analysis. By annotating information, it was preliminarily revealed that *OsNAC113* is involved in multiple biological processes, such as “plant hormone signaling pathway”, “MAPK signaling pathway”, “amino acid transport and metabolism”, “carbohydrate transport and metabolism”, and “lipid transport and metabolism and replication recombination and repair”, and responds to high-salt stress. Based on metabolomics sequencing results, knocking out *OsNAC113* resulted in changes in various important plant biosynthetic pathways, including flavonoid biosynthesis, plant hormone signaling pathways, and ABC transporters, which responded to various abiotic stress conditions and improved the salt tolerance of *OsNAC113*.

## Data Availability

This study has provided all generated and analyzed data as requested. The sequence data of MutMap have been deposited in the GSA under accession number subCRA050633. The original contributions presented in the study are included in the article/Supplementary Materials, further inquiries can be directed to the corresponding authors.

## Supplementary Materials

The following supporting information can be downloaded at: https://www.mdpi.com/article/10.3390/plants14233673/s1, Table S1: Analysis of the promoter sequence. Table S2: Bioinformatics analysis online software. Table S3: Protein interaction relationship table. Table S4: Detection results predicted as off target sites. Table S5: Primers used. Figure S1: *OsNAC113* encodes protein phosphorylation sites. Figure S2: *OsNAC113* encoded protein hydrophilicity/hydrophobicity distribution curve. Figure S3: Prediction of transmembrane domain of *OsNAC113* encoded protein. Figure S4: Arginine and proline metabolic pathways.
