# Supplementary material for: Knockout of the OsNAC113 Transcription Factor Causes High Salt Resistance in Rice
Source: Plants (Basel). 2025 Dec 2;14(23):3673. doi: 10.3390/plants14233673 (PMC12694509; doi:10.3390/plants14233673)

Supplymental FigureS1

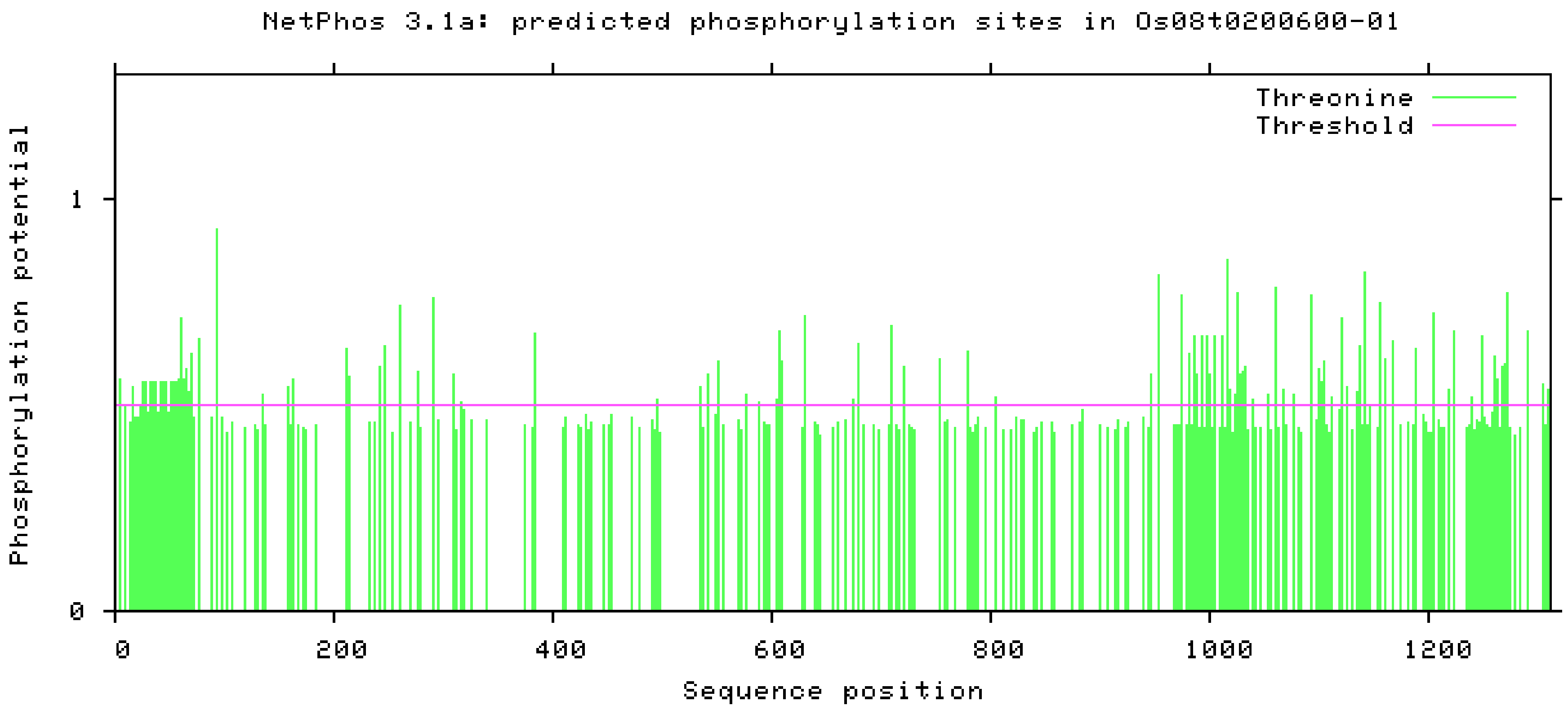

Supplymental FigureS2

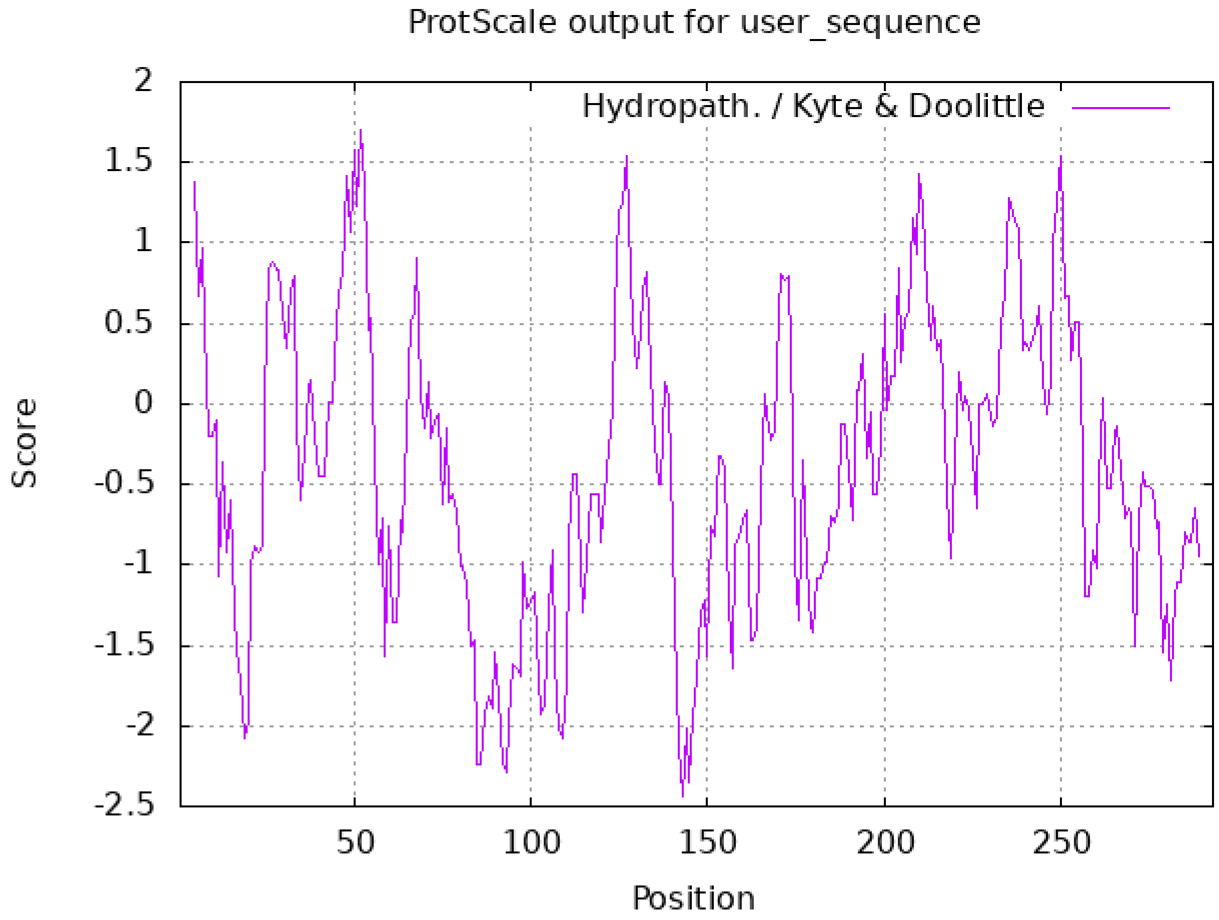

Supplymental FigureS3

```
# Os08t0200600-01 Length: 293
# Os08t0200600-01 Number of predicted TMHs:  0
# Os08t0200600-01 Exp number of AAs in TMHs: 0.01001
# Os08t0200600-01 Exp number, first 60 AAs:  0.00733
# Os08t0200600-01 Total prob of N-in:        0.01703
Os08t0200600-01 TMHMM2.0        outside      1   293
```

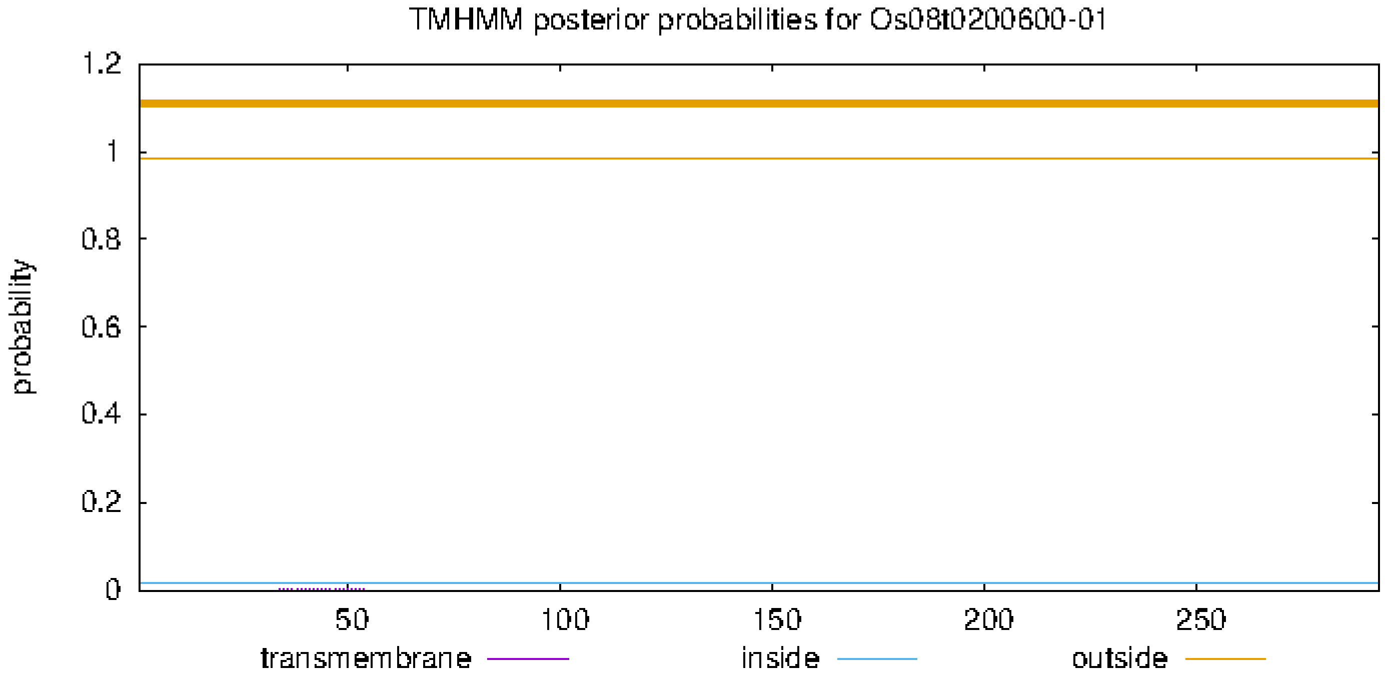

Supplymental FigureS4

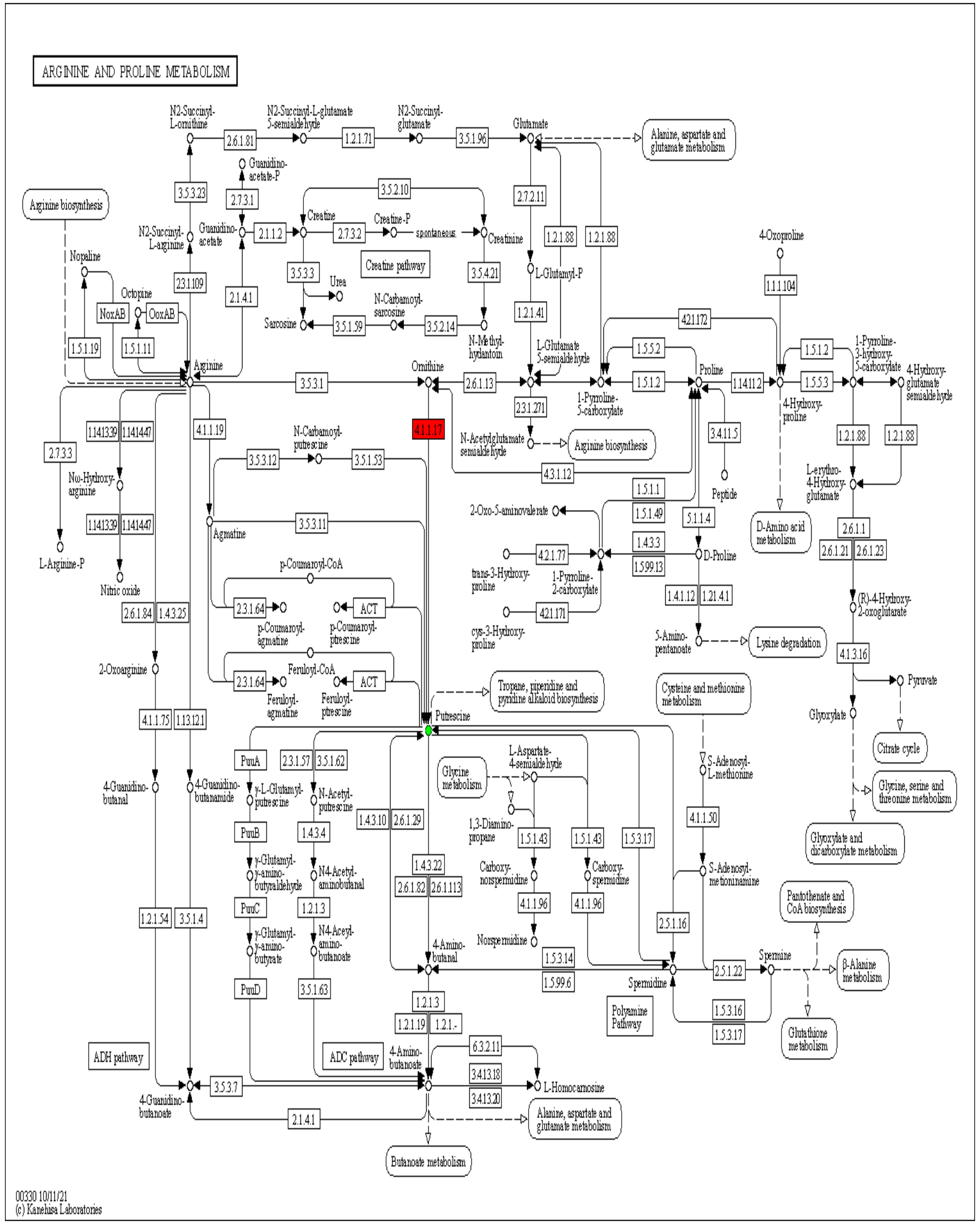

Supplement: Supplementary file 1 [file plants-14-03673-s001.zip › plants-3965508-supplementary figures.pdf]
