# Supplementary material for: Knockout of the OsNAC113 Transcription Factor Causes High Salt Resistance in Rice
Source: Plants (Basel). 2025 Dec 2;14(23):3673. doi: 10.3390/plants14233673 (PMC12694509; doi:10.3390/plants14233673)
Supplement: Supplementary file 1 [file plants-14-03673-s001.zip › plants-3965508-supplementary tables.pdf]

plants

MDPI

Supplementary Material

Supplementary Table S1. Analysis of the promoter sequence

Factor or Site Name	Signal Sequence	Reference	Number of binding sites
WRKY71OS	TGAC	The binding site of rice WRKY71, a transcriptional repressor of the gibberellin signaling pathway, affects abscission.	14
ACGTATERD1	ACGT	The ACGT sequence (–155 to –152) is required for yellowing-induced expression of erd1 (early dehydration response) in Arabidopsis.	8
ABRELATERD1	ACGTG	The ABRE-like sequence (–199 to –195) required for the expression of erd1 (early dehydration response) induced by yellowing in Arabidopsis.	1
MYBCORE	CNGTTR	Participate in regulating genes in Arabidopsis that respond to water stress.	11
MYB2CONSENSUSAT	YAACKG	The MYB recognition site was found in the promoter of the dehydration response gene rd22 in Arabidopsis.	3
MYB2AT	TAACTG	Involved in the dehydration signaling pathway in *Arabidopsis thaliana*	1
MYCCONSENSUSAT	CANNTG	The MYC recognition site was found in the promoter of the dehydration response gene rd22 in Arabidopsis	8
DOFCOREZM	AAAG	The core site required for Dof protein binding in maize.	14
GT1GMSCAM4	GAAAAA	The GT-1 motif is present in the promoter of the soybean (Glycine max) CaM isomer SCaM-4; Plays a role in pathogen and salt-induced SCaM-4 gene expression.	5

Supplementary Table S2 Bioinformatics analysis online software

Software/database(website)	Content of analysis
EXPASy<br>https://web.expasy.org/protparam/	Prediction of physical and chemical properties
Netphos3.1<br>https://services.healthtech.dtu.dk/services/NetPhos-3.1/	Phosphorylation site
TMHMM2.0<br>http://www.cbs.dtu.dk/services/TMHMM/	Transmembrane domain prediction
ProtScale<br>https://web.expasy.org/protscale/	Analysis of protein hydrophilicity and hydrophobicity
http://pfam.xfam.org/	Structure domain prediction

Supplementary Table S3. Protein interaction relationship table.

gene1	gene2	protein1	protein2	combined _score
Os01t0112600-01	Os04t0598900-01	4530.OS01T0 112600-01	4530.OS04T0598900-01	822
Os01t0123900-01	Os12t0569500-01	4530.OS01T0 123900-01	4530.OS12T0569500-01	843
Os01t0124000-01	Os03t0289800-01	4530.OS01T0 124000-01	4530.OS03T0289800-01	810
Os01t0186900-01	Os06t0683400-02	4530.OS01T0 186900-02	4530.OS06T0683400-02	795
Os01t0644200-01	Os11t0454000-01	4530.OS01T0 644200-01	4530.OS11T0454000-01	761
Os01t0705200-01	Os11t0454000-01	4530.OS01T0 705200-01	4530.OS11T0454000-01	716
Os01t0743500-01	Os06t0246500-01	4530.OS01T0 743500-01	4530.OS06T0246500-01	986
Os01t0743500-01	Os08t0434300-01	4530.OS01T0 743500-01	4530.OS01T0829800-01	994
Os02t0288100-01	Os04t0604300-01	4530.OS02T0 288100-01	4530.OS04T0604300-01	781
Os02t0703600-01	Os03t0790700-01	4530.OS02T0 703600-01	4530.OS03T0790700-01	875
Os02t0791500-02	Os08t0526100-01	4530.OS02T0 791500-01	4530.OS08T0526100-01	866
Os02t0791500-02	Os08t0445700-02	4530.OS02T0 791500-01	4530.OS08T0445700-01	961
Os03t0180800-01	Os04t0395800-01	4530.OS03T0 180800-01	4530.OS04T0395800-01	714
Os03t0221200-01	Os10t0399700-00	4530.OS03T0 221200-01	4530.OS10T0399700-00	972
Os03t0221200-01	Os10t0399200-00	4530.OS03T0 221200-01	4530.OS10T0399200-00	972

Os03t0265500-01	Os04t0395800-01	4530.OS03T0265500-01	4530.OS04T0395800-01	993
Os03t0860100-01	Os05t0381400-01	4530.OS03T0860100-01	4530.OS05T0381400-01	757
Os04t0386700-01	Os04t0610600-01	4530.OS04T0386700-01	4530.OS04T0610600-01	798
Os05t0573300-01	Os11t0126400-00	4530.OS05T0573300-01	4530.OS11T0126400-00	959
Os07t0209100-01	Os07t0687900-01	4530.OS07T0209100-01	4530.OS07T0687900-01	981
Os07t0511400-01	Os12t0113600-01	4530.OS07T0511400-01	4530.OS12T0113600-01	761
Os07t0511400-01	Os12t0113700-00	4530.OS07T0511400-01	4530.OS12T0113700-00	761
Os08t0445700-02	Os10t0521000-01	4530.OS08T0445700-01	4530.OS10T0521000-01	991
Os12t0113600-01	Os12t0113700-00	4530.OS12T0113600-01	4530.OS12T0113700-00	761

Supplementary Table S4 Detection results predicted as off target sites

Target	Chromosome	Position	Direction	Mismatches	examinedlinesNumbe	off-targets Number
CGGGGtGGAtGGGTGTtCGGGGG	chr3	12977006	+	3	9	0
CGtGGAGGAGGGGcGTgCGGGGG	chr7	9348783	+	3	9	0
CGaGGAGGAGaGGTGTgCGGCGG	chr4	93070	+	3	9	0
CGcGGAGGAGGcGTGgACGGGGG	chr4	22662050	+	3	9	0
gGGGGAGGAGGaGgGTACGGGGG	chr5	22271296	+	3	9	0

CGGGGtGGAGcGGTGcACGGTGG	chr1	25830597	-	3	9	0
CGGGGAGGAGGGGcGacCGGCGG	chr1	40442825	-	3	9	0
CGGGGAGGAGGGGTGccCGaAGG	chr2	13988107	+	3	9	0
CGGGGAGGAGGGGaGaAgGGGGG	chr2	16979689	+	3	9	0
CGGGGAGGAGGGGgaaACGGAGG	chr2	18145862	-	3	9	0

Supplementary Table S5 Primers used

Primer Name	forward primer（5′→ 3′）	reverse primer（5′→ 3′）
OsNAC113-RT	CCATCACCCGCCGCTCCTC	TGGGTCATGCACGAGTTC
Off-target 1	AAAGCACGAAGAGCCACCACA	TGGGAAACTCCGGCACGAC
Off-target 2	CGCTGCCTCTTTCGTCTC	ATGCCCTGCCAGCCAACT
Off-target 3	CCTCTTCCCGCTAGACTTT	CTTGCCAGACAGGATGTTC
Off-target 4	TGTTGATGATGGCGAGAA	ATCAGCCACATTGGAAGG
Off-target 5	TTGCCTGGCTATGATACTCT	TGTGCTTAGTGCCTTCCTC
Off-target 6	ACACCGACATGGAGGTTGA	AGAGGCGAAGAGGAAGCA
Off-target 7	CGGCAGGGTCAAGAGTTCG	CGTCTCGCTGGAGGAGTTCG
Off-target 8	TGGACTCGCTGGACCAGAAC	CGGTGTAGGGCTGGAACG
Off-target 9	GCAGACGCAGACCAAGAAGAA	GGCGGCAGAGGAAAGAGC
Off-target10	GCCACCATCGAGAAGACGAAGCG	AGCAGGCCGAACTTGCCGTAG
